# Quantifying circulating cell-free DNA in humans

**DOI:** 10.1038/s41598-019-41593-4

**Published:** 2019-03-26

**Authors:** Romain Meddeb, Zahra Al Amir Dache, Simon Thezenas, Amaëlle Otandault, Rita Tanos, Brice Pastor, Cynthia Sanchez, Joelle Azzi, Geoffroy Tousch, Simon Azan, Caroline Mollevi, Antoine Adenis, Safia El Messaoudi, Philippe Blache, Alain R. Thierry

**Affiliations:** 1 IRCM, Institute of Research in Oncology of Montpellier, Montpellier, France; 2 INSERM, U1194 Montpellier, France; 3 University of Montpellier, Montpellier, France; 4 Regional Institute of Cancer of Montpellier, Montpellier, France; 5 Biometry Unit, Regional Institute of Cancer of Montpellier, Montpellier, France; 6 Digestive Oncology Department, Regional Institute of Cancer of Montpellier, Montpellier, France

## Abstract

To our knowledge, this is the first comprehensive study on the influence of several pre-analytical and demographic parameters that could be a source of variability in the quantification of nuclear and mitochondrial circulating DNA (NcirDNA and McirDNA). We report data from a total of 222 subjects, 104 healthy individuals and 118 metastatic colorectal cancer (mCRC) patients. Approximately 50,000 and 3,000-fold more mitochondrial than nuclear genome copies were found in the plasma of healthy individuals and mCRC patients, respectively. In healthy individuals, NcirDNA concentration was statistically influenced by age (*p* = *0.009*) and gender (*p* = *0.048*). Multivariate analysis with logistic regression specified that age over 47 years-old was predictive to have higher NcirDNA concentration (OR = 2.41; *p* = *0.033*). McirDNA concentration was independent of age and gender in healthy individuals. In mCRC patients, NcirDNA and McirDNA levels were independent of age, gender, delay between food intake and blood collection, and plasma aspect, either with univariate or multivariate analysis. Nonetheless, ad hoc study suggested that menopause and blood collection time might have tendency to influence cirDNA quantification. In addition, high significant statistical differences were found between mCRC patients and healthy individuals for NcirDNA (*p* < *0.0001*), McirDNA (*p* < *0.0001*) and McirDNA/NcirDNA ratio (*p* < *0.0001*). NcirDNA and McirDNA levels do not vary in the same way with regards to cancer vs healthy status, pre-analytical and demographic factors.

## Introduction

**Figure 1:**
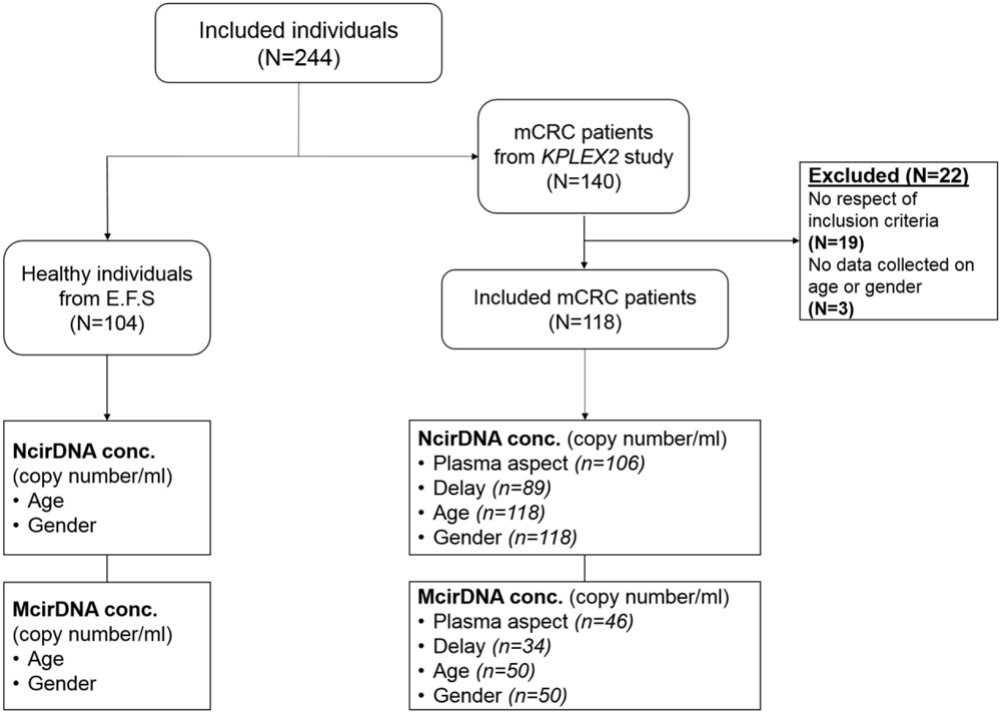
Flow chart of the study.

Since Mandel and Metais discovered the presence of nucleic acids in serum in the 1940s^1^, different studies have reported elevated levels of circulating DNA (cirDNA) in the blood of patients suffering from various diseases^2–6^, especially cancer^7–10^. Despite scant early consideration, interest in the feasibility of cirDNA analysis has increased exponentially, over the last decade, among researchers working on a large range of disorders. CirDNA was first clinically implemented in prenatal diagnosis of sex-determination and pregnancy-associated disorders by assaying fetal DNA in maternal plasma^11–13^. The main sources of cirDNA are cell death, either by necrosis or apoptosis, and active release by viable cells, including exocytosis and NETosis^14,15^. Note, cirDNA may derive from either nuclear (NcirDNA) or mitochondrial DNA (McirDNA). To date, research and development of cirDNA analysis has focused on the qualitative rather than the quantitative information provided. For example, cirDNA analysis is now clinically validated for detecting specific sequences or mutations to guide the oncologist toward the most appropriate treatment. CirDNA analysis is also performed for prenatal and embryo-culture genetic testing. There have been several years of intensive studies validating cirDNA quantitation in different clinical scenarios, including sepsis, transplant recipients and immune disorders. CirDNA quantification is also now taken into consideration in oncology as, it was recently shown that, the level of mutant cirDNA is useful for following-up cancer patients to detect minimal residual disease and to monitor response to therapy and disease recurrence. Although total cirDNA levels were first examined in the early phase of cirDNA research and development, it is now not considered as a single biomarker because of its lack of specificity. Nevertheless, all cirDNA analysis relies on the optimal quantification of total amount of cirDNA. Biological biomarkers should be highly dynamic, and their diagnostic performance may vary depending on internal and external changes. The clinical efficacy of cirDNA will require the identification and the control of various patient-related confounders that may affect its measurement^16^. Human cellular aging is usually marked by senescence and cell death. Similarly, it often features a phenomenon, originally called “Inflamm-aging”, that induces a chronic or low-grade inflammatory state^17–20^. Indeed, it was demonstrated that tissue damage and a pro-inflammatory environment increase release of cirDNA into the blood stream^14^. Additionally, we may speculate that sex-based differences, such as genetic dissimilarity and steroid hormones levels, could cause differences in cirDNA concentration between men and women. Likewise, blood component concentrations may vary with the circadian clock and upon food intake and potentially influencing cirDNA concentration^21^. Despite outstanding research in the field of cirDNA, relatively few studies have examined the effect of pre-analytical and demographic parameters as sources of intra- and inter-individual variability in cirDNA levels. There is currently no single operating procedure and there are relatively few clinical guidelines in the literature^22–25^. This study aims first at defining a framework for cirDNA analysis, to harmonize NcirDNA and McirDNA quantification and to explore potential sources of variability that could cause interpretation errors. Previous studies already documented various pre-analytical limitations and specified conditions for cirDNA analysis, including specific collection tubes, and specific plasma isolation and extraction protocols, including storage condition variables and limits on the number of freeze-thaw cycles^22–24,26^. Here, we study the influence of various pre-analytical (plasma aspect, delay between blood collection and last food intake) and demographic variables such age and gender in NcirDNA and McirDNA concentration determination, from plasma of 104 healthy donors and 118 mCRC patients (Fig. 1). In addition, we compared McirDNA and NcirDNA levels in healthy individuals and mCRC patients. Some others pre-analytical conditions such as blood collection tubes, blood collection time and blood stability were examined *ad hoc* study on healthy volunteers.

## Results

### Simultaneous quantification of NcirDNA and McirDNA

We used serial dilutions of genomic DNA and of mitochondrial plasmid DNA to validate a reproducible, linear and sensitive assay to quantify, both nuclear and mitochondrial, circulating DNA (NcirDNA and McirDNA). The NcirDNA assay is based upon the qPCR detection of a sequence of the *KRAS* gene and it can detect one copy of nuclear *KRAS* gene per 6 microliters of plasma (1 nuclear Genome Equivalent per 12 µL). The McirDNA assay can detect down to one copy of the mitochondrial *MT-CO3* gene per 1.7 microliter of plasma (1 mitochondrial Genome Equivalent per 1.7 µL). Note, the targeted MT-CO3 gene sequence was selected as being not mutated in the mitochondrial genome of cancer patients. Copy number calculation is performed by using a specific equation that eliminates bias in the nuclear or mitochondrial DNA calibration curves to allow simultaneous calculation of their real relative proportions. For example, in the cohort of 104 healthy individuals, we found a median NcirDNA and McirDNA plasma concentration of 1.64 × 10^3^ and 8.32 × 10^7^ copies/mL, respectively, corresponding to 5.43 and 1.36 ng/mL of plasma.

### Comparison between NcirDNA and McirDNA levels

**Figure 2:**
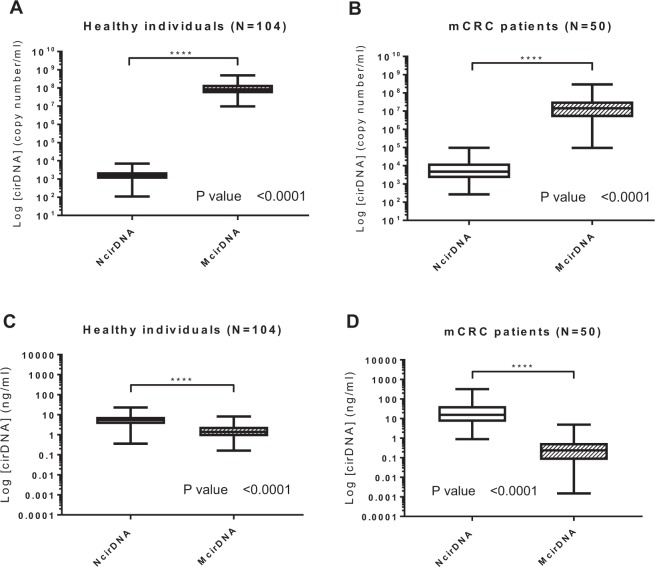
Respective values of NcirDNA and McirDNA plasma concentration. Boxplot analysis of cirDNA concentrations from healthy individuals (N = 104) (**A**,**C**) and mCRC patients (N = 50) (**B**,**D**). Values are expressed either as copy number/ml (**A**,**B**) or as ng/ml (**C**,**D**). CirDNA concentration was determined as described in Materials and Methods. Boxplot represent median with min to max of values and Mann-Whitney U test was performed for comparison. A probability of ≤0.05 was considered to be statistically significant; *p ≤ 0.05, **p ≤ 0.01, ***p ≤ 0.001, ****p ≤ 0.0001.

We compared NcirDNA and McirDNA concentration, in healthy individuals and mCRC patients groups, expressed either in copy number/ml and ng/ml of plasma. A supplementary table summarizes all quantification data and comparative tests in detail (Supplementary Table S1). There was a highly statistical difference between NcirDNA and McirDNA concentration expressed in copy number/ml, in healthy individuals (Fig. 2A; *Mann-Whitney U test, P value* < *0.0001*) and equally in mCRC patients (Fig. 2B; *Mann-Whitney U test, P value* < *0.0001*). We next compared McirDNA and NcirDNA concentration expressed in ng/ml, in healthy individuals and mCRC patients groups. Here, also, we observed a considerable statistical difference between NcirDNA and McirDNA concentrations in healthy individuals (Fig. 2C; *Mann-Whitney U test, P value* < *0.0001*) and mCRC patients (Fig. 2D; *Mann-Whitney U test, P value* < *0.0001*). McirDNA concentration is significantly higher than NcirDNA concentration when measured in units of copy number/ml, and conversely, the NcirDNA concentration was significantly higher than the McirDNA concentration when measured in ng/ml, either in healthy individuals and mCRC patients.

### Effect of age and gender on cirDNA concentrations in healthy individuals

#### NcirDNA

**Figure 3:**
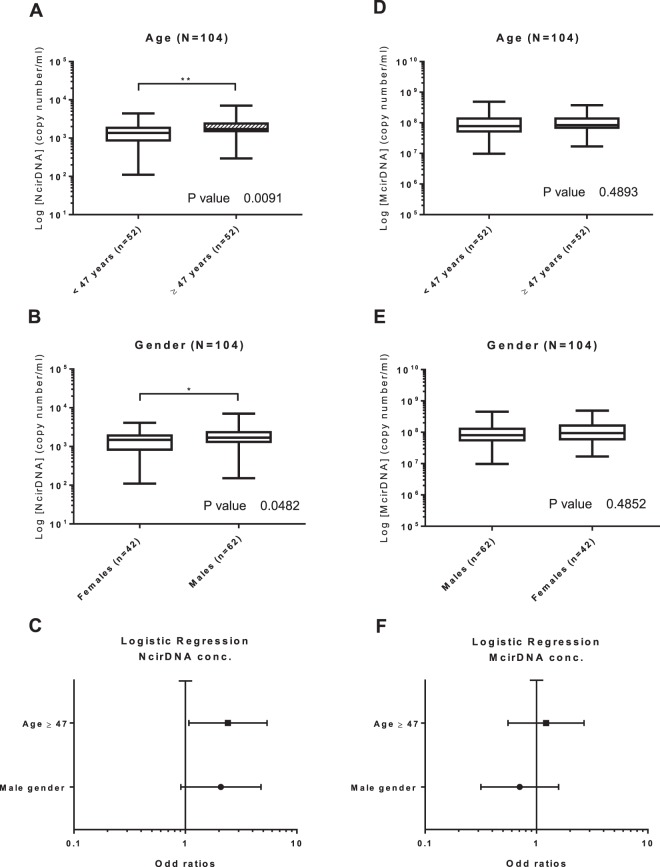
Influence of age and gender on cirDNA concentration in healthy individuals. Boxplot analysis of cirDNA concentration extracted from healthy individuals (N = 104), with regards to age (**A,D**) and gender (**B**,**E**). (**C**,**F**) Multivariate analysis representations. NcirDNA (**A**–**C**) and McirDNA (**D**–**F**) concentrations are expressed in copy number/ml of plasma. Boxplot represent median with min to max of values. Mann-Whitney U test was performed for univariate analysis and logistic regression was performed for multivariate analysis. Odds ratio (OR) with 95% confidence intervals (CIs) are represented. A probability of ≤0.05 was considered to be statistically significant; *p ≤ 0.05, **p ≤ 0.01, ***p ≤ 0.001, ****p ≤ 0.0001.

**Age (n = 104):** We dichotomized the healthy individuals cohort in two groups around the median age, which was 47 (Table 1). The median NcirDNA copy number in the <47 years-old group (n = 52) and in the ≥47 years-old group (n = 52) were 1.36 × 10^3^ and 1.73 × 10^3^ copies/ml, respectively. A statistical difference was found between young and older healthy individuals groups (Fig. 3A; *Mann-Whitney U test, P value* = *0.009)*.

**Table 1:** Characteristics of healthy individuals (N = 104) and mCRC patients (N = 118).

Patient’s characteristics					
Healthy individuals (N = 104)			mCRC patients (N = 118)		
**Age (years)**					
Mean	45		Mean	65	
Median	47		Median	65	
(min-max)	(18–69)		(min-max)	(22–91)	
**Gender**					
Males	62	59,6%	Males	68	57,6%
Females	42	40,4%	Females	50	42,4%
TOTAL	99		TOTAL	118	
**Males (N)**					
Mean age	45		Mean age	65	
Median age	47		Median age	65	
(min-max)	(19–69)		(min-max)	(34–88)	
**Females (N)**					
Mean age	44		Mean age	65	
Median age	45		Median age	67	
(min-max)	(18–63)		(min-max)	(22–91)

**Gender (n = 104):** The median NcirDNA copy number in the healthy male group (n = 62) and in the group of healthy females (n = 42) were 1.69 × 10^3^ and 1.48 × 10^3^ copies/ml, respectively. This difference in the NcirDNA copy number between healthy males and females was statistically significant (Fig. 3B; *Mann-Whitney U test, P value* = *0.048*).

**Multivariate analysis:** Logistic regression analysis including age and gender specified that age over 47 years-old was predictive to have higher NcirDNA concentration (Fig. 3C; OR = 2.41, *P* = *0.033*). Results of multivariate analysis and Odds ratios (OR) with 95% confidence intervals (CIs) are summarized in Supplementary Table S2A.

#### McirDNA

**Age (n = 104):** The median McirDNA copy number in the <47 (n = 52) and in the ≥47 year-old group (n = 52) were 7.77 × 10^7^ and 8.40 × 10^7^ copies/ml, respectively. There was no statistical difference in the McirDNA copy number between these groups (Fig. 3D; *Mann-Whitney U test, P value* = *0.489*).

**Gender (n = 104):** The median McirDNA copy number in the healthy male group (n = 62) and in the group of healthy women (n = 42) were 8.03 × 10^7^ and 9.39 × 10^7^ copies/ml, respectively. No statistical difference in McirDNA copy number was observed between healthy males and females (Fig. 3E; *Mann-Whitney U test, P value* = *0.485*).

**Multivariate analysis:** Logistic regression analysis including age and gender confirmed no statistically significant difference between studied groups (Fig. 3F). Results of multivariate analysis and Odds ratios (OR) with 95% confidence intervals (CIs) are summarized in Supplementary Table S2B.

### Effect of various parameters on cirDNA concentrations in mCRC patients

#### NcirDNA

**Figure 4:**
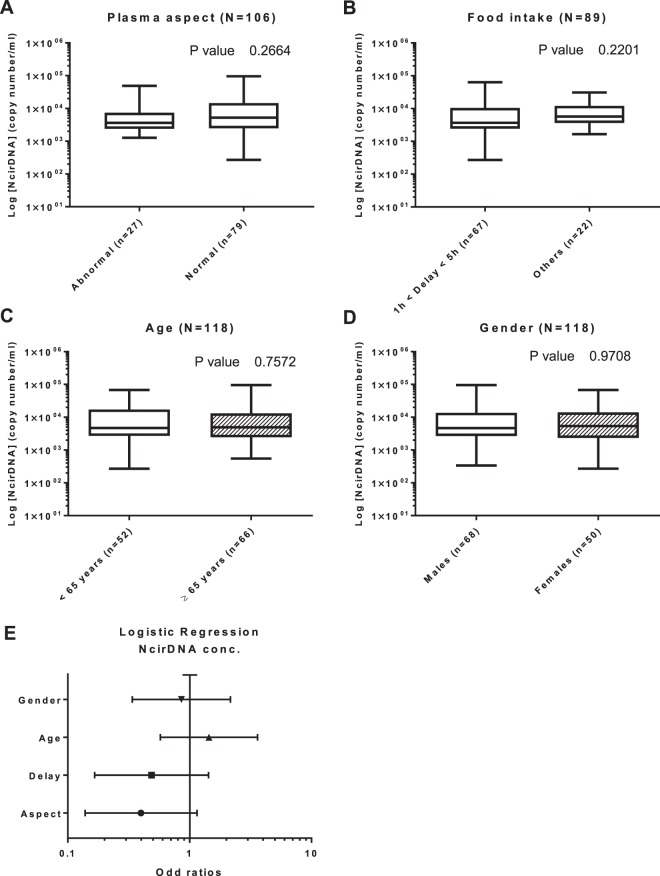
Influence of various factors on NcirDNA concentration in mCRC patients. Boxplot analysis of NcirDNA concentration extracted from mCRC patients (N = 118), with regards to plasma aspect (**A**); delay between blood collection and last food intake (**B**); age (**C**) and gender (**D**). (**E**) Multivariate analysis representation. Boxplot represent median with min to max of values. Mann-Whitney U test was performed for univariate analysis and logistic regression was performed for multivariate analysis. Odds ratio (OR) with 95% confidence intervals (CIs) are presented. A probability of ≤0.05 was considered to be statistically significant; *p ≤ 0.05, **p ≤ 0.01, ***p ≤ 0.001, ****p ≤ 0.0001.

**Plasma aspect (n = 106):** We first compared two groups, 27 abnormal plasmas (icteric and/or opaque plasmas) and 79 normal plasmas. There was no statistical difference in NcirDNA levels between abnormal plasmas and normal plasmas groups (Fig. 4A; *Mann-Whitney U test; P value* = *0.266*). However, the median NcirDNA amount determined in abnormal plasmas group was slightly lower than in normal plasmas (3.62 × 10^3^ vs 5.22 × 10^3^ copies/ml).

**Delay between blood collection time and last food intake (n = 89):** We then compared two groups, “1 h < delay < 5 h” (n = 69) and “other delays” (n = 20). There was no statistical difference in NcirDNA levels between “1 h < delay < 5 h” and “other delays” (Fig. 4B; *Mann-Whitney U test; P value* = *0.220*). Nonetheless, the “1 h < delay < 5 h” group showed lower median NcirDNA amount than the group “other delays” (3.68 × 10^3^ vs 5.72 × 10^3^ copies/ml).

**Age (n = 118):** We also dichotomized the mCRC cohort into two groups around the median age of 65 (Table 1). The median NcirDNA copy number in the <65 year-old group (n = 52) and in the ≥65 year-old group (n = 66) were 4.73 × 10^3^ and 4.94 × 10^3^ copies/ml, respectively. No statistical difference was found (Fig. 4C; *Mann-Whitney U test, P value* = *0.757*). A comparative study using the same cut-off for both healthy and mCRC cohorts as the median age of all individuals tested here (N = 222, median age = 56 years) confirmed the statistical difference between young (N = 79) and older (N = 25) healthy individuals groups (*Mann-Whitney U test, P value* = *0.0026)*, and also the no statistical difference between young (N = 25) and older (N = 93) mCRC groups (*Mann-Whitney U test, P value* = *0.913*) (Supplementary Fig. S1A,B).

**Gender (n = 118):** The median NcirDNA copy number in the mCRC males (n = 68) and in and females (n = 50) mCRC patients were 4.65 × 10^3^ and 5.40 × 10^3^ copies/ml, respectively. No statistical difference was found (Fig. 4D; *Mann-Whitney U test, P value* = *0.971*).

**Multivariate analysis:** Logistic regression analysis including all the parameters (plasma aspect, delay, age and gender) confirmed no statistical significant results (Fig. 4E). Results of multivariate analysis and Odds ratios (OR) with 95% confidence intervals (CIs) are summarized in Supplementary Table S2C.

#### McirDNA

**Figure 5:**
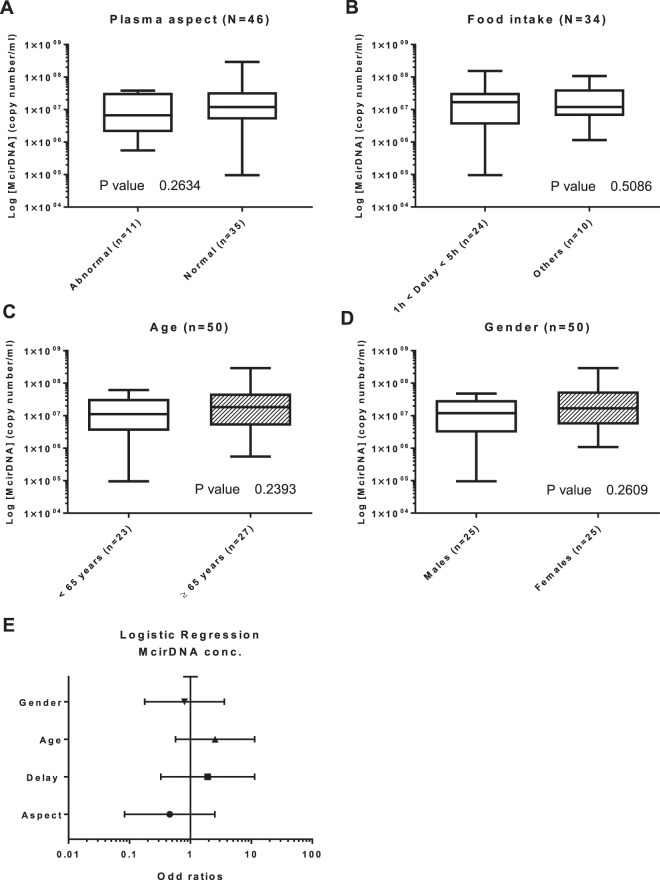
Influence of various factors on McirDNA concentration in mCRC patients. Boxplot analysis of McirDNA concentration extracted from mCRC patients (N = 50), with regards to plasma aspect (**A**); delay between blood collection and last food intake (**B**); age (**C**) and gender (**D**). (**E**) Multivariate analysis representation. Boxplot represent median with min to max of values. Mann-Whitney U test was performed for univariate analysis and logistic regression was performed for multivariate analysis. Odds ratio (OR) with 95% confidence intervals (CIs) are presented. A probability of ≤0.05 was considered to be statistically significant; *p ≤ 0.05, **p ≤ 0.01, ***p ≤ 0.001, ****p ≤ 0.0001.

**Plasma aspect (n = 46):** We next compared the abnormal plasmas (n = 11) and normal plasmas (n = 35) groups. There was no statistical difference in NcirDNA levels between abnormal and normal plasmas groups (Fig. 5A; *Mann-Whitney U test; P value* = *0.263*). However, the median McirDNA amount determined in abnormal plasmas was slightly lower than in normal plasmas (6.64 × 10^6^ vs 1.19 × 10^7^ copies/ml).

**Delay between day-time of blood draw and the last food intake (n = 34):** There was no statistical difference in McirDNA copy number between the “1 h < delay < 5 h” group (n = 25) and the “other delay” group (n = 9) (Fig. 5B; *Mann-Whitney U test; P value* = *0.509*). The “1 h < delay < 5 h” group showed higher median NcirDNA amount than the group “other delay” (1.68 × 10^7^ vs 1.19 × 10^7^ copies/ml).

**Age (n = 50):** The median McirDNA copy number in the < 65 year-old (n = 23) and ≥ 65 year-old (n = 27) groups were 1.12 × 10^7^ and 1.84 × 10^7^ copies/ml, respectively. There was no statistical difference in the McirDNA copy number between the groups (Fig. 5C; *Mann-Whitney U test, P value* = *0.240*).

**Gender (n = 50):**The median McirDNA copy number in the mCRC males group (n = 25) and in the group of mCRC women (n = 25) were 1.19 × 10^7^ and 1.68 × 10^7^ copies/ml, respectively. We did not found any statistical difference in the McirDNA copy number between mCRC males and females (Fig. 5D; *Mann-Whitney U test, P value* = *0.261*).

**Multivariate analysis:** Logistic regression analysis including all the parameters (plasma aspect, delay, age and gender) confirmed no statistically significant results (Fig. 5E). Results of multivariate analysis and Odds ratios (OR) with 95% confidence intervals (CIs) are summarized in Supplementary Table S2D.

### Comparing cirDNA levels between mCRC patients and healthy individuals

#### NcirDNA

**Figure 6:**
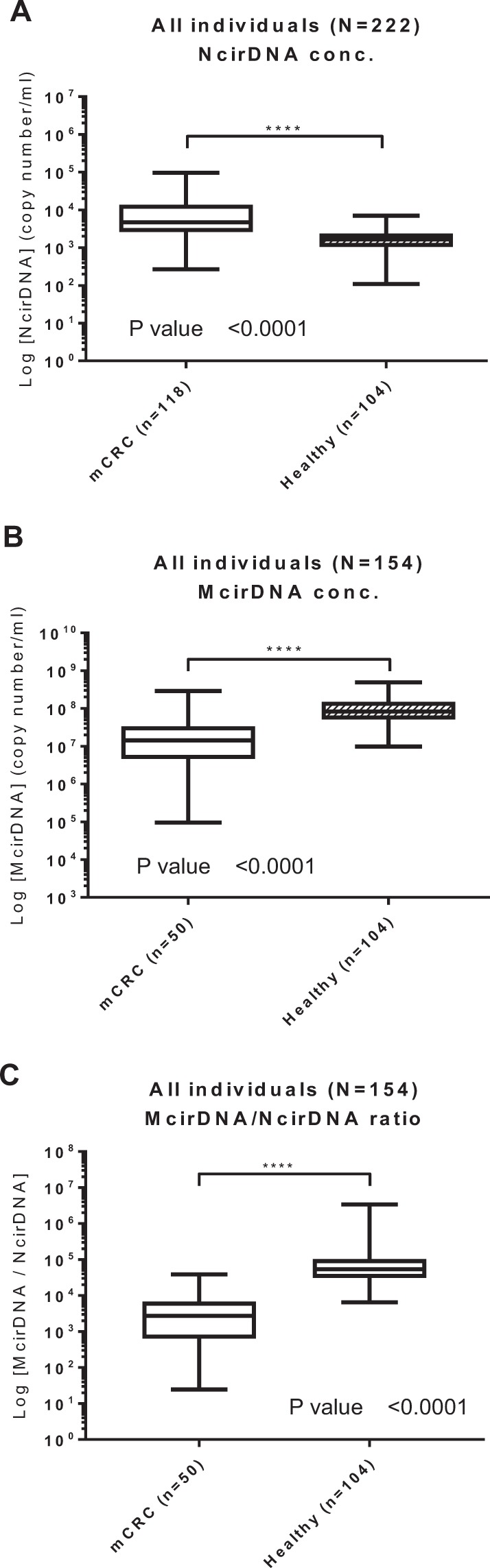
Biomarker capacity of respective NcirDNA and McirDNA concentration for discriminating healthy individuals and mCRC patients. The cohort was dichotomized in two populations (mCRC patients and healthy individuals). Boxplot analysis of the amount of NcirDNA (**A**) and McirDNA (**B**) extracted from plasma of all individuals (N = 222 and N = 154, respectively). (**C**) Boxplot analysis of the McirDNA/NcirDNA ratio of all individuals (N = 154). The boxplots represent medians with min to max of values and Mann-Whitney U test was performed for comparison. A probability of ≤0.05 was considered to be statistically significant; *p ≤ 0.05, **p ≤ 0.01, ***p ≤ 0.001, ****p ≤ 0.0001.

We compared the median NcirDNA amount between healthy individuals (n = 104) and mCRC patients (n = 118). The median NcirDNA concentration in healthy individuals and mCRC patients was 1.64 × 10^3^ and 4.73 × 10^3^ copies/ml, respectively, revealing a significant difference between healthy individuals and mCRC patients (Fig. 6A; *Mann-Whitney U test, P value* < *0.0001*).

#### McirDNA

Next we compared the median McirDNA amount between healthy individuals (n = 104) and mCRC group (n = 50). The median McirDNA concentration in healthy individuals and mCRC patients was statistically different at 8.32 × 10^7^ and 1.44 × 10^7^ copies/ml, respectively (Fig. 6B; *Mann-Whitney U test, P value* < *0.0001*).

#### McirDNA/NcirDNA ratio

Finally we compared the median McirDNA/NcirDNA ratio between healthy individuals (n = 104) and mCRC patients (n = 50). The median McirDNA/NcirDNA ratio in healthy individuals and mCRC patients were statistically different at 5.41 × 10^4^ and 2.70 × 10^3^ copies/ml, respectively (Fig. 6C; *Mann-Whitney U test, P value* < *0.0001*). The median McirDNA/NcirDNA ratio was 20-fold higher in the healthy individuals group than in the mCRC patients group. We did the same comparison using McirDNA and NcirDNA concentrations expressed in ng/ml and we obtained the same results (Supplementary Fig. S2, *Mann-Whitney U test, P value* < *0.0001)*.

### CirDNA stability in whole blood samples of healthy volunteers

NcirDNA concentration in blood collected in EDTA tubes increased by more than two-fold and three-fold respectively, at days 2 and 5 after blood collection (Supplementary Fig. S3A*)*. In contrast, there was no difference between days 0, 2, 5 and 7 following blood collection in Cell-Free DNA BCT® STRECK (BCT) tubes (Supplementary Fig. S3B). Note, median values at day 0 are similar in plasma from blood collected in EDTA or BCT tubes. In addition, The McirDNA concentration in blood collected in EDTA tubes approximately increased more than 12-fold and 27-fold respectively, at days 2 and 5 after blood collection (Supplementary Fig. S3C).

### Effect of the blood collection time on cirDNA levels in healthy volunteers

#### NcirDNA

In EDTA tubes at 9.00 AM (fasted state), 12.00 PM (2 hours after breakfast), 3.00 PM (2 hours after lunch) and 6.00 PM, the median NcirDNA copy numbers were 1500, 480, 715 and 709 copies/ml respectively. An additional figure shows this in more detail (Supplementary Fig. S4A,E). Taking the four healthy individuals together, the median NcirDNA concentration strongly changed from the fasted state to three hours after breakfast. Median NcirDNA concentration was slightly higher at the earliest collection time in BCT tubes, over the same time-course of the day, at 874, 562, 728 and 313 copies/ml, respectively. An additional figure shows this in more detail (Supplementary Fig. S4B,F). Altogether our data from both collection methods showed that NcirDNA content in healthy individuals declined from 9.00 AM to 12.00 PM then plateaued up to the 6.00 PM collection time. An additional figure shows this in more detail (Supplementary Fig. S4C,D,G,H).

#### McirDNA

In EDTA tubes from healthy individuals, the median NcirDNA copy numbers were unchanged over the time course at 1.05, 1.25, 1.17 and 1.26 million copies/ml. An additional figure shows this in more detail (Supplementary Fig. S5A,B). No clear difference was found when comparing McirDNA amounts collected at the various collection time points. Note, plasma appearance become opaque at 6.00 PM. An additional picture illustrates this observation (Supplementary Fig. S6).

## Discussion

Here, we present a comprehensive study on the quantification of nuclear and mitochondrial cirDNA in the plasma of healthy individuals and a homogenous cohort of mCRC patients. To our knowledge, this work is the first to address altogether the influence of various preanalytical, analytical and demographical factors on NcirDNA and McirDNA levels in a large set of individuals (n > 200). Solid tumor mass is composed of a variety of cells, mostly consisting of malignant/cancer, stromal, endothelial and immunological cells. In order to avoid any confusion in nomenclature, we define as ‘tumor cells’ all the cells composing the tumor mass. The concentration values presented in this study correspond to total cell-free DNA in plasma, either of nuclear or mitochondrial origin. Our observations are summarized in Table 2 and Supplementary Table S3.

**Table 2:** Summary of the observations made on the influence of demographical factors.

Demographical considerations	Group	N	In accordance with previous works
**NcirDNA concentration**			
A statistical difference between males and females	Healthy	104	Catarino R, *et al*.^63^
No statistical difference between males and females	mCRC	118	Hao TB, *et al*.^31^
A statistical difference with regard to age	Healthy	104	Jylhävä J, *et al*.^35^
No statistical difference with regard to age	mCRC	118	Van der Drift MA, *et al*.^30^
Statistical increase in mCRC patients as compared to healthy individuals	Healthy mCRC	104 118	Bedin C, *et al*.^64^
**McirDNA concentration**			
No statistical difference between males and females	Healthy mCRC	104 50	New observation
No statistical difference with regard to age	Healthy mCRC	104 50	Jylhävä J, *et al*.^35^ New observation
Statistical decrease in mCRC patients as compared to healthy individuals	Healthy mCRC	104 50	New observation
**McirDNA vs NcirDNA concentration**			
Necessity of independently quantifying NcirDNA and McirDNA			
About 50,000-fold more McirDNA copy number as compared to NcirDNA in healthy individuals	Healthy	104	New observation
About 3,000-fold more McirDNA copy number as compared to NcirDNA in cancer patients	mCRC	50	New observation
About 20-fold more McirDNA/NcirDNA ratio in healthy individuals as compared to mCRC patients	Healthy mCRC	104 50	New observation

### Total cirDNA levels

This study design relies on the analytical performance of the assay. We used here a qPCR-based method with an unmatched combination, of reproducibility, sensitivity and specificity for quantifying plasma cirDNA (see Methods section). Furthermore, our assay benefits from a clinically-validated optimal pre-analytic process that we previously set up for plasma preparation^22^; and adapted from Chiu *et al*., DNA extraction and sample handling^27^. The accuracy of the cirDNA concentration measurement in this study is supported by two assessments: (i) total cirDNA concentration by targeting a *KRAS* sequence was routinely controlled by quantifying a *BRAF* sequence. In addition, this control quality enable to detect and exclude sample with loss of heterozygosity (LOH) or gene amplification which have been reported in CRC patients^28^. Moreover, since *KRAS* amplification is an infrequent event in CRC (0.67%)^28^, this level will not modify our observations or the values described in our manuscript; and (ii) the study of cirDNA measurement under Poisson law distribution revealed single copy detection of nuclear cirDNA.

Respective NcirDNA and McirDNA proportions inverted when the plasma cirDNA concentrations were calculated according to copy number or mass (Fig. 2A,C). For example, data obtained from 104 healthy individuals revealed that median McirDNA plasma concentration was approximately 50,000-fold higher than median NcirDNA plasma concentration in terms of copy number/mL (99.998% McirDNA and 0.002% NcirDNA), corresponding to approximately 4-fold lower in terms of ng/ml (25.0% McirDNA and 75.0% NcirDNA). This highlights the much lower size of the mitochondrial genome (16569 bp) than the nuclear genome (about 3 × 10^9^ bp), and also the high number of mitochondrial genome copies within a cell. Each human cell, depending on its type, contains a number of mitochondria ranging from 500 to 2,000 and each mitochondria holds between 2 and 10 mitochondrial DNA molecules. Therefore, each cell may contain approximatively 1,000 to 20,000 mitochondrial DNA molecules and only one nuclear genome equivalent, which is consistent with our results. Our method of calculation, described here, appears as the most rigorous means to simultaneously quantify mitochondrial and nuclear DNA concentrations. A higher coefficient of variation was routinely observed for mitochondrial than for nuclear DNA, despite the higher analytical signal for McirDNA copy number. The median concentration levels we found here appear to be similar to the average values observed in several studies^27,29^. In addition, performance of our assay support the accuracy of the values presented throughout this study such as 5.43 and 1.36 ng/mL in healthy subject plasma of N- and McirDNA, respectively. Considering the significant percentage of McirDNA (i.e.: 25% in healthy subjects) among total cirDNA mass existing in plasma, we propose to always specify the origin of cirDNA when examining total circulating WT DNA.

### Influence of age and gender on NcirDNA levels in healthy individual plasma

Most studies found no relationship between NcirDNA levels and any demographic parameters, such as age and gender, in healthy individuals^30–32^. A few studies showed opposite observations^33–35^. Here, we observed an influence of age on NcirDNA plasma concentration in the healthy individual cohort as a whole (*p* = *0.009*). Our data also revealed a statistical difference in NcirDNA amount between healthy males and females (*p* = *0.048*). We performed logistic regression for multivariate analysis including age and gender and showed that age ≥ 47 was predictive of a high NcirDNA concentration (OR = 2.41, *p* = *0.033*). This observation is in accordance with a study by Jylhävä *et al*. that consisted of 12 nonagenarian women (age > 90 years) and 11 healthy control female (22 < age < 37 years) that showed a higher concentration of NcirDNA in nonagenarians than in control women^33^. The authors explained this increase in the amount of cirDNA with age, as the accentuation of a senescence phenomenon and cell death, caused by an inflammation associated with age and even by decreased clearance and phagocytic capacity. Zhong *et al*. showed a significant increase in total plasma cirDNA concentration in women over 60, compared to younger women, which appears to be consistent with Jylhävä’s study. We may speculate that menopause could be an explanation of the statistically higher NcirDNA concentration in healthy males as compared to healthy females (p = 0.048), whereas no difference between mCRC males and females was observed (p = 0.971). This speculation is based on two observations: (i) Median menopausal age in the European population is 51 years-old; 10–15% of women go into menopause before the age of 45, and globally 90–95% reach menopause by 55 years of age. By applying these categories of age to our women cohorts, we observed no statistical difference and no tendency between mCRC women <45, 45–54 and ≥55 year-old (*Kruskal-Wallis rank test, P value* = *0.592*) while we showed a statistical difference between healthy women <45, 45–54 and ≥55 year-old, with a concentration gradient: 1275, 1440 and 2610 median copy number, respectively (*Kruskal-Wallis rank test, P value* = *0.026*); and (ii) CRC females are at a high risk of chemotherapy-induced menopause or menstrual disorders like amenorrhea or a decrease of steroid hormone levels^36,37^, and metachronous mCRC patients may attain a menopausal state earlier. An additional figure shows these results in more detail (Supplementary Fig. S7).

### Influence of various parameters on NcirDNA levels in mCRC patients plasma

Despite the large number of studies that aimed at determining if age and gender might influence NcirDNA levels, no clear results have been demonstrated^30–32,38^. There are discrepancies in the literature with regards to the influence of age and gender in patient populations suffering from various types of cancers. These discrepancies could result from use of serum, or pre-analytical or analytical factors. Note, a study by Hohaus *et al*. showed that patients with Hodgkin and non-Hodgkin’s lymphoma over 60 year-old (n = 142) had higher levels of cirDNA in plasma than younger patients (*p* = *0.018*)^38^. Conversely, our data showed no statistical influence of age (*p* = *0.757*) and gender (*p* = *0.971*) on NcirDNA concentration in mCRC patients and these results was confirmed with a multivariate analysis using logistic regression. We also demonstrated no influence of pre-analytical factors like plasma aspect (*p* = *0.266*) or delay between last food intake and blood collection (*p* = *0.220*). On the other hand, our results also confirm those previously published by our laboratory^25^ and by many teams^39,40^, namely that NcirDNA concentration is significantly higher cancer patients than in healthy individuals (*p* < *0.0001*), whether male or female. For more than a decade it was suggested that total NcirDNA could be a cancer biomarker^25,41,42^. However, previous attempts to apply total cirDNA quantity as a screening test for cancer lacked a strong statistical demonstration^43^. High standards, with regard to pre-analytical factors and quantification, could lead to its use as one marker, among other, for tumor burden.

### McirDNA levels in plasma

Relatively few reports have quantitatively analyzed McirDNA and there are discrepancies among them. In one study there was no significant difference between young and aged healthy subjects^35^. For Pinti *et al*. however, McirDNA concentration would increase with age^18^. In this study, McirDNA content was analyzed in 831 plasma samples from subjects with different healthy status, aged from 1 to 104 years; McirDNA content significantly increased after fifty years-of-age and it peaked in nonagenarians. Elevated McirDNA levels might help maintain the low-grade chronic inflammation that is common in elderly individuals. With regards to McirDNA level in cancer patients, Mengel-From *et al*. measured McirDNA copy number in blood cells from 1,067 subjects aged 18 to 93 and conversely, observed a tendency for lower mitochondrial DNA copy number with advanced age^44^. These findings are consistent with other studies, performed on different types of tissue, such as skeletal muscle and pancreatic islets^45,46^. This age-related tissue-specific depletion of cellular mitochondrial DNA could lead to a proportional reduction of McirDNA copy-number in plasma. Inversely, there was no association between serum McirDNA levels and demographic parameters (age/gender) in urological malignancies^47,48^. Likewise, in breast cancer, there was no significant difference in McirDNA content in blood samples of stage I patients with respect to their age^49^. Our data revealed no influence of age and gender on McirDNA concentration, either in plasma of healthy individuals or mCRC patients. These results were confirmed in multivariate analysis. We also reported no significant influence of plasma aspect (p = 0.263) and delay between last food intake and blood collection (p = 0.509). Nonetheless, our data revealed a significant higher McirDNA than NcirDNA concentration in plasma, whether for mCRC patients, healthy individuals, male or female, and regardless of the age of the subject. Note, while median NcirDNA concentration is much higher in mCRC patients than in healthy individuals, median McirDNA concentration is conversely lower in mCRC patients, revealing a proportionally lower McirDNA release from cancer cells. This might be explained by the fact that cancer cells, in comparison to healthy cells, may have, among other differences, fewer mitochondria per cell and less DNA within their mitochondria^34,50^. However, this is still controversial and explanation of our striking observation is under active investigation in our team. Nevertheless, we may speculate that the McirDNA/NcirDNA ratio might have some power in discriminating healthy individuals from cancer patients. McirDNA/NcirDNA ratio is undergoing clinical validation as potential biomarker for tumor burden or diagnosis in a large study involving broader scope of cancer patients with various malignancies and stages. In light of the high copy number of McirDNA and its tendency to be mutated in cancer^51^, our observations confirm the gradual acceptance of McirDNA as a new potent diagnostic and prognostic biomarker for many solid tumors^52^.

### Blood stability for cirDNA plasma assessment

As previously reported^23,53^, NcirDNA concentration determined from blood collected in EDTA tubes increased with time highlighting release of genomic DNA resulting from blood cell lysis when stored at room temperature or +4 °C and consequently to contamination of cell-derived DNA. Note, whole blood stored in EDTA tubes at +4 C° showed no change in cirDNA concentration for up to one day suggesting their potential use within this time period (data not shown)^23,53^. We propose routine clinical analyses use plasma stored in EDTA tubes for up to 6 hours, given the uncertainty of maintaining the temperature of samples in the course of blood processing, as we earlier described^54^. Conversely, BCT tubes appeared to conserve blood cell integrity, since no DNA concentration increase was observed up to 7 days following blood collection^23,55^. Thus, BCT tubes maintain the true cirDNA concentration and are good tools to conserve/stabilize blood for optimal quantification of NcirDNA for up to 7 days following collection. Cell-preserving tubes greatly allows postal shipment of whole-blood within this time period and it allows interventional analysis as well as enabling clinical centers that lack lab facilities to immediately prepare plasma. While being cheaper by themselves, use of EDTA tubes necessitates plasma preparation within a short time frame and immediate subsequent storage under frozen conditions until analysis requiring costly shipment when plasma originate from a single patient. We first reported that McirDNA concentration determined from blood collected in EDTA tubes strongly increased with time. We may assume that this results as well blood cell lysis and blood cell-derived mitochondrial DNA contamination.

### Effect of blood collection time on cirDNA plasma concentration

There is currently no indication in the literature on the optimal time for blood collection when analyzing cirDNA. Our data seem to indicate that NcirDNA median levels are 2- to 3-fold higher at 9.00 AM, which is the earliest time-point examined, compared to later blood-collection time-points (12.00, 3.00 and 6.00 PM) when the NcirDNA level stabilizes. Decrease from 9.00 AM to 12.00 PM might be explained by the postprandial effect of the breakfast being taken at 10.00 AM. This hypothesis is supported by several observations. First, we reported that NcirDNA plasma levels in blood collected between one and five hours after food intake were lower than in blood collected on patients under fasting conditions. It was previously showed that plasma triglyceride increased one hour after food intake, peaked ≈3 hours after intake of a test meal and baseline values were restored back to initial values after 5 hours^56^. Second, NcirDNA concentration was lower in opaque than in non-opaque plasmas. Multivariate analysis including age, gender, plasma aspect and delay between food intake and blood collection revealed no statistical influence but abnormal plasma aspect showed a clear tendency to have lower NcirDNA concentration (OR = 0.399; *p* = *0.089*). These observations are all consistent with postprandial effects. Food intake with high lipid content may result in hyperlipidemia which can be characterized by opaque plasma and high triglyceride concentrations. However, despite the large examined cohort data, we cannot state that postprandial is the explanation since no statistical difference was found. This may be due to various factors: (i) blood triglyceride levels largely depend on fat distribution and body weight, lifestyle choices, and also genetic factors^57^; (ii) there were considerable within- and between-subject variations in non-fasting plasma triglycerides^58^; and (iii) the subjects had a chronic illness, mCRC. We speculate that the postprandial effect could occur because the presence of lipids or proteins may interfere with DNA extraction yield from plasma. We cannot exclude the possibility that NcirDNA levels depend on circadian clocks and metabolism, resulting in more elevated concentration in the morning. Moreover, we cannot exclude the possibility that other metabolic changes during fasting/feeding alter cirDNA yield. Nevertheless, our data suggest that fasting blood samples should be included when studying or clinically examining cirDNA to improve its diagnostic performance, especially when low mutation frequency in cancer patients or prenatal testing is considered. Note, in addition to opaque plasma, we remarked on various occasions that, icteric plasma had aberrant cirDNA concentration when qPCR was the analytical method. Therefore, we propose observation of icteric, hemolytic and opaque plasma as criteria of blood sample exclusion.

### Limitations of the study

While the study was carried out with statistically sufficient number of subjects to support the observations, the *ad hoc* study is limited by the low number of tested individuals since *ad hoc* study experiments are cumbersome and time-consuming. Thus, blood stability and blood collection time was only carried out on a few number of healthy volunteers (n = 5) and not on mCRC patients. This not allowed us to provide statistical analysis while the results showed clear tendencies. A specific study should be performed to statistically confirm these results on a larger cohort of healthy volunteers as well as mCRC patients. Although we routinely experienced that abnormal plasmas resulted in lowering cirDNA concentration values, we cannot fully discriminate the implication of postprandial effects like triglycerides serum level, to the possible involvement of biological changes dues to the circadian rhythm. In order to definitively address this issue, it would be interesting to compare NcirDNA levels at 9.00 AM and 12.00 AM, with and without breakfast, in order to determine the impact of food intake and circadian rhythm, respectively. In addition, the influence of the menopause on cirDNA concentration with regard to our observations of gender, age and pathological status is only speculative. A specific study on the difference of NcirDNA concentration between postmenopausal and premenopausal healthy females, as well as the difference between premenstrual and postmenstrual young women, should be performed to definitively address this issue. Conclusions drawn here in respect to cancer plasma samples should be restricted to mCRC patients and extension to other malignancies or even to localized disease is speculative.

In conclusion, the levels of mitochondrial and nuclear circulating DNA differently vary with regards to pre-analytical and demographic factors. Those variables should be taken into consideration when evaluating cirDNA analysis in clinical setting and perhaps in the future clinical practice when cirDNA quantification is directly or indirectly used as a biomarker. In addition, our study highlights the potential for combining the analysis of NcirDNA and McirDNA since examining their respective levels may have diagnostic value.

## Methods

### Patients

Blood samples collected from 104 healthy donors were provided by the Etablissement Français du Sang (E.F.S), the blood transfusion center of Montpellier (Convention EFS-PM N° 21PLER2015-0013). Blood samples collected by the E.F.S were analyzed (virology, serology, immunology, blood numeration). If an anomaly is detected, the sample is ruled out and the donor is warned then by mail. NcirDNA concentration and McirDNA copy number were determined for all healthy individuals. Data on age and gender were collected for all healthy individuals. mCRC patients data are taken from a study comparing the detection of *KRAS* exon 2 and *BRAF V600E* mutations by circulating DNA (cirDNA) analysis to conventional detection by tumor tissue analysis^59^. This study (KPLEX2) was performed and presented under the STARD criteria. 140 patients have been included in 11 clinical centers in France, over a period of 12 months. Eligible patients were male or female, aged ≥18 years, with a proven histologically mCRC, a measurable disease as defined by response evaluation criteria in solid tumors (RECIST v1.1) and untreated by radiotherapy or chemotherapy in the last 4 weeks before inclusion. There is no possibility that cirDNA from mCRC patients can be affected by therapy since eligible patients were untreated by radiotherapy or chemotherapy in the last 4 weeks before blood collection (inclusion criteria). Written consent was obtained from the part of all patients. Inclusion criteria were described previously^59^. 19 were excluded from analysis for no respect of the inclusion criteria (due to various inclusion criteria) and 3 were excluded due to lack of data collected on age or gender. NcirDNA analysis was performed on 118 patients and McirDNA analysis on 50/118 mCRC patients. While study on age and gender effect were carried out on all the included mCRC patients for NcirDNA (N = 118), cohort patient number varied when studying plasma aspect and delay for NcirDNA, or age and gender for McirDNA because of two main reasons: non-reported information for delay and plasma aspect for to NcirDNA and insufficient plasma volume needed to carry out supplementary analysis for McirDNA. All data on age and gender were collected. Plasma aspect (normal, abnormal: opaque or/and icteric) was noted for 106/118 mCRC patients and delay between time of blood draw and the last food intake was informed for 89/118 mCRC patients.

### Ethics approval and consent to participate

Blood samples collected from 104 healthy donors were provided by the Etablissement Français du Sang (E.F.S), the blood transfusion center of Montpellier (Convention EFS-PM N° 21PLER2015-0013). Plasma samples from mCRC patients were obtained from the Kplex2 study registration number EUDRACT 2016-001490-33 with ethic committee approval (“Comité de Protection des Personnes”, Nimes, France). All methods were performed in accordance with the relevant guidelines and regulations. The study obtained informed consent from all participants for the study.

### Blood stability

7 samples for each healthy donor were collected at 9.00 a.m. on Day 0 (fasted state): 3 with EDTA K2 tubes and 4 with Cell-Free DNA BCT® STRECK tubes. Each tube was processed as we early described, at day 0, 2 and 5 for EDTA tubes and day 0, 2, 5 and 7 for BCT tubes. EDTA tubes were stored at +4 °C and BCT tubes were conserved at room temperature before isolation. Healthy individual N°1 (HI1) and N°2 (HI2) are 57 year-old and 29 year-old men, respectively, both with no known disease. Healthy individual N°3 (HI3), N°4 (HI4) and N°5 (HI5) are 25 year-old, 24 year-old and 28 year-old women, respectively, both with no known diseases. NcirDNA analysis for HI1 was not performed due to clotting in BCT tubes.

### Blood collection time

4 blood collection times were defined 9.00 a.m. (fasted state), 12.00 p.m. (2 hours after breakfast), 3.00 p.m. (2 hours after lunch) and 6.00 p.m. (4 hours after lunch). One EDTA K2 and one Cell-Free DNA BCT® STRECK tube per day-time were collected for each donor. Healthy individual N°6 (HI6) and N°7 (HI7) are 29 year-old and 27 year-old men, respectively, both with no known diseases. Healthy individual N°8 (HI8) and N°9 (HI9) are 28 year-old and 30 year-old women respectively, both with no known diseases. Each donors took the same meal during breakfast: one butter croissant, one chocolate croissant and one coffee with sugar; and for the lunch: a dish of tomato rice with sausage, bread and one coffee with sugar.

### Sample characteristics and preparation

Samples were collected and treated in accordance with a pre-analytical guideline previously established by our group^22^. In summary, blood was collected in EDTA K3 tubes and plasma was isolated within 2 hours. The isolation technique consist of a double centrifugation. Initially, tubes were centrifuged for 10 minutes at 4 °C and 1,200 g in a Heraeus Multifuge LR centrifuge. The supernatant was collected while carefully avoiding the buffy-coat. The second centrifugation was conducted for 10 minutes at 4 °C and 16,000 g. The supernatant was transferred to 1.5 ml tubes before performing the extraction of cirDNA or being stored at −20 °C. CirDNA extraction was performed with the Qiagen Blood Mini kit, following all steps of the protocol. In all, 0.2 to 1 ml of plasma was extracted in several successive passes on a column. The final elution volume was 80 to 130 µl and eluates were frozen at −20 °C prior to analysis by qPCR. Freeze-thaw cycles should be avoided to reduce the phenomenon of cirDNA fragmentation and the extracts are not kept longer than 3 months at −20 °C.

### Q-PCR analysis

CirDNA analysis was performed by a qPCR technique developed in our laboratory, and clinically validated previously^60^. The method is based on an innovative design of short amplicons (60–100 bp ± 10 bp) targeting a wild-type sequence of the gene, (here the *KRAS* nuclear gene and the mitochondrial Cytochrome oxidase III gene*, MT-CO3)*. Quantification of this amplicon gives an estimation of the total NcirDNA and McirDNA concentration, respectively. For the quantification of NcirDNA, we amplified of a 67 bp-length sequence of the KRAS gene with the following primers: forward (5′ CCTTGGGTTTCAAGTTATATG 3′) and reverse (5′ CCCTGACATACTCCCAAGGA 3′). For McirDNA, we amplified a 67 bp-length sequence of the cytochrome oxidase sub-unit 3 mitochondrial gene with the following primers: forward (5′ GACCCACCAATCACATGC 3′) and reverse (5′ TGAGAGGGCCCCTGTTAG 3′). These primers were designed using the Primer 3 software according to the following requirements: (i) *T*_m_ ranging from 50 to 64 °C; (ii) GC-content between 40 and 60%; (iii) size from 18 to 23 NT; (iv) amplicon size ranging from 60 to 100 bp. We performed local-alignment analyses with the BLAST program to confirm the specificity of the designed primers. All sequences were checked for self- or inter-molecular annealing with nucleic-acid-folding software (Mfold and oligoAnalyzer 1.2). Oligonucleotides were synthesized and HPLC-purified by Eurofins (Ebersberg, Germany) and quality control of the oligonucleotides was performed by MALDI-TOF. For all analyses, negative controls and standard curves were used. All tests are performed in triplicate with 5 µl of DNA extract in a 25 µl reaction volume, on a CFX96 instrument using CFX manager software (Bio-Rad). This method (qPCR, primer design, program) and technical validation have been described previously^25^. The mCRC patient’s blood samples were excluded if the total cirDNA concentration, due to a problem of pre-analytic treatment or even for unknown reasons, was below a quality threshold.

The DNA concentration quality threshold was 3 ng/mL (about 900 copies/mL) for cancer patients. Note, this value corresponds to about the half of the median concentration found for healthy individuals (N = 104, median NcirDNA concentration = 5.43 ng/mL of plasma corresponding to 1645 copies/mL). In addition to quantifying cirDNA by targeting two different sequences on two different chromosomes, an experiment based on Poisson law distribution showed the accuracy of our cirDNA amount measurement (Supplementary Fig. S8). It should be noted that our Q-PCR systems enable the detection of a single genome copy (Supplementary Table S4), and that we have previously shown that targeting a DNA sequence of the same size or longer than the input DNA fragment produced a similar PCR yield^61^. The measurement of the total cirDNA concentration by targeting a *KRAS* sequence was routinely controlled by quantifying a *BRAF* sequence. Control quality is acceptable when the *KRAS*-based value is 1.3 to 1.8-fold higher than that of the *BRAF*-based value when using the reported Q-PCR primer systems; otherwise, a second analysis is performed. Note, *BRAF* analysis data from 33 healthy individuals were not available. In addition, we excluded 26 patient plasmas (17 mCRC and 9 healthy) in which the *KRAS*/*BRAF* ratio was over 3 or below 0.5. Data revealed that the *KRAS*-based concentration value was positively correlated with the *BRAF*-based concentration values in the 62 healthy individuals (Spearman analysis; r = 0.762, *P value* < *0.0001*) and in the 101 mCRC patients (Spearman analysis; r = 0.882, *P value* < *0.0001*) (Supplementary Fig. S9). We already addressed this issue in our previous report (Spearman analysis; r = 0.966, *P value* < *0.001*)^25^.The *KRAS* and *BRAF* genes are monogenic and poorly amplified in both healthy and cancer individuals^62^. Supplementary section figures present NcirDNA concentration determined from targeting *BRAF* with using a primer set of similar size. Data revealed that the same observations could be made: the NcirDNA amount, as determined using *BRAF* sequence targeting, is statistically different in healthy (N = 62) and mCRC individuals (N = 101) (Supplementary Fig. S10; Mann-Whitney U test, *P value* < *0.0001*), and fully correlates with our observation based on *KRAS* sequence targeting. We may therefore indicate that amplification of the *KRAS* gene will not have any influence on the observations and conclusions made in this study. Valtorta *et al*. detected *KRAS* amplification in 7/1,039 (0.67%) evaluable CRC specimens, demonstrating that *KRAS* amplification is an infrequent event in CRC^28^. Thus this level will not modify significantly the observations or values described in our manuscript.

### CirDNA calibration assay

#### NcirDNA

A genomic DNA extract from human wild-type *KRAS* colorectal cells was used for the NcirDNA calibration assay. Initial genomic DNA solution concentration and purity were determined by measuring optic density at λ = 260 nm, 230 nm and 280 nm, with an Eppendorf BioPhotometer® D30. Starting genomic DNA concentration was adjusted to 1800 pg/µl for the first dilution point, according to optic density measurement at λ = 260 nm. A qPCR standard curve was obtained by 6 successive dilutions of the vector solution (1800, 180, 45, 20, 10 and 5 pg/µl). The standard curve was used to determine the NcirDNA concentration of the mCRC patients and healthy individuals and calculate the NcirDNA copy number per milliliter of plasma.

#### McirDNA

A 3382-pb human ORF vector with a 786-pb *MT-CO3* insert was obtained from ABM good® (accession no.YP_003024032) and used for the McirDNA calibration assay. Initial vector solution concentration and purity were determined by measuring optic density at λ = 260 nm, 230 nm and 280 nm, with an Eppendorf BioPhotometer® D30. Starting vector concentration was adjusted at 1800 pg/µl for the first dilution point, according to optic density measurement at λ = 260 nm. A qPCR standard curve was obtained by 6 successive dilutions of the vector solution (1800, 180, 45, 20, 10 and 5 pg/µl). The standard curve was used to determine the McirDNA concentration of the mCRC patients and healthy individuals and calculate the McirDNA copy number per milliliter of plasma.

### CirDNA copy number calculation

#### NcirDNA

NcirDNA copy number per milliliter of plasma, in all analyses, was determined with the following calculation:

$$Qnuclear=(\frac{c}{3,3})\ast (\frac{V\acute{e}lution}{Vplasma})$$

Q_nuclear_ is the NcirDNA copy number per milliliter, c is the NcirDNA concentration (pg/µl) determined by qPCR targeting the nuclear *KRAS* gene sequence and 3.3 pg is the human haploid genome mass. V_elution_ is the volume of cirDNA extract (µl) and V_plasma_ is the volume of plasma used for the extraction (ml).

#### McirDNA

McirDNA copy number per milliliter of plasma, in all analyses, was determined with the following calculation:

$$Qmito=(\frac{c\ast Na}{2\ast MW\ast Lvector})\ast (\frac{V\acute{e}lution}{Vplasma})$$

Q_mitochondrial_ is the McirDNA copy number per milliliter, ‘c’ is the McirDNA mass concentration (g/µl) determined by a qPCR targeting the mitochondrial *MT-CO3* gene. N_A_ is Avogadro’s number (6.02 * 10^23^ molecules per mole), L_vector_ is the plasmid length (nucleotides) and MW is the molecular weight of one nucleotide (g/mol). V_elution_ is the elution volume of cirDNA extract (µl) and V_plasma_ is the volume of plasma used for the extraction (ml).

### Statistical analysis

For continuous variables, median and range were computed. To investigate their associations with the biologic parameters, univariate statistical analyses were performed using Mann-Whitney U test or Kruskal-Wallis rank test for continuous variables. Moreover, multivariate analyses were carried out using logistic regressions, with a stepwise selection procedure, to investigate known predictive. Odds ratio (OR) with 95% confidence intervals (CIs) are presented. The power of analysis was reduced due to all patients did not have measurements for all variables. All P values reported are two sided. A probability of ≤0.05 was considered to be statistically significant; *p ≤ 0.05, **p ≤ 0.01, ***p ≤ 0.001, ****p ≤ 0.0001. Statistical analysis was performed using the STATA 13.1 software (Stata Corporation, College Station, TX).

## Supplementary information

Supplementary information

## Data Availability

The datasets used and/or analyzed during the current study are available from the corresponding author on reasonable request.
