# Supplementary material for: Quantifying circulating cell-free DNA in humans
Source: Sci Rep. 2019 Mar 26;9:5220. doi: 10.1038/s41598-019-41593-4 (PMC6435718; doi:10.1038/s41598-019-41593-4)
Supplement: Supplementary file 1 — Supplementary information [file 41598_2019_41593_MOESM1_ESM.docx]

**Quantifying circulating cell-free DNA in humans**

**Romain Meddeb^1,2,3,4^, Zahra Al Amir Dache^1,2,3,4^, Simon Thezenas^1,2,3,4,5^, Amaëlle Otandault^1,2,3,4^, Rita Tanos^1,2,3,4^, Brice Pastor^1,2,3,4^, Cynthia Sanchez^1,2,3,4^, Joelle Azzi^1,2,3,4^, Geoffroy Tousch^1,2,3,4^, Simon Azan^1,2,3,4^,** **Caroline Mollevi^1,2,3,4,5^, Antoine Adenis^1,2,3,4,6^, Safia El Messaoudi^1,2,3,4^, Philippe Blache^1,2,3,4^, and Alain R. Thierry^1,2,3,4*^**

**^^**

**Supplementary Figure S1: Influence of age on NcirDNA concentration.** Boxplot analysis of NcirDNA concentration as determined by targeting *BRAF* sequence with using a primer set of similar size, in healthy individuals (*A*) and mCRC patients (*B*). Boxplot represent median with min to max of values and Mann-Whitney U test was performed for comparison. A probability of ≤ 0.05 was considered to be statistically significant; * p ≤ 0.05, ** p ≤ 0.01, *** p ≤ 0.001, **** p ≤ 0.0001.

**Supplementary Figure S2: Biomarker capacity of respective NcirDNA and McirDNA concentration for discriminating healthy individuals and mCRC patients.** Boxplot analysis of cirDNA concentration expressed in ng/ml. Boxplot represent median with min to max of values and Mann-Whitney U test was performed for comparison. A probability of ≤ 0.05 was considered to be statistically significant; * p ≤ 0.05, ** p ≤ 0.01, *** p ≤ 0.001, **** p ≤ 0.0001.

**Supplementary Figure S3: CirDNA stability in full blood.** Boxplot analysis of NcirDNA *(A, B)* and McirDNA *(C)* concentration in plasma collected in EDTA tubes *(A, C, N=5)* and BCT tubes *(B, N=4).* Plasma preparation and cirDNA extraction were carried out following blood collection for Day 0 or delayed at Day 2, Day 5 or Day 7. CirDNA concentration was determined in triplicate and expressed as copy number/ml plasma. Boxplot represent median with min to max of values.


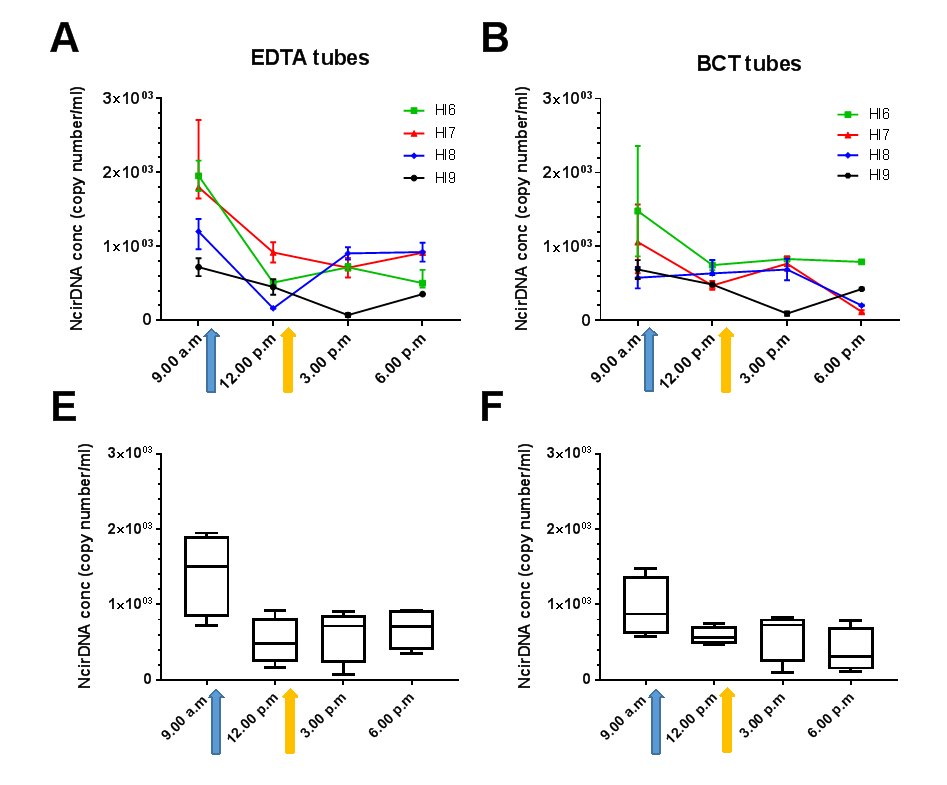


**Supplementary Figure S4: Effect of the blood collection time on NcirDNA amount in healthy volunteers.** Variation of NcirDNA concentration expressed as copy number/ml for 4 healthy donors, collected in EDTA *(A, E)* and BCT *(B, F)* tubes at various time points during the same day. Blue and yellow arrows indicate breakfast and lunch respectively. Composition of food intake is described in Material and Methods. *(A, B, C, D, G and H)* NcirDNA concentration values determined from blood collected in *EDTA* and *BCT* tubes were plotted for each healthy individuals (HI6, HI7, HI8 and HI9). Data represent triplicate median values with range. *(E* and *F)* Boxplot represent median with min to max of values.


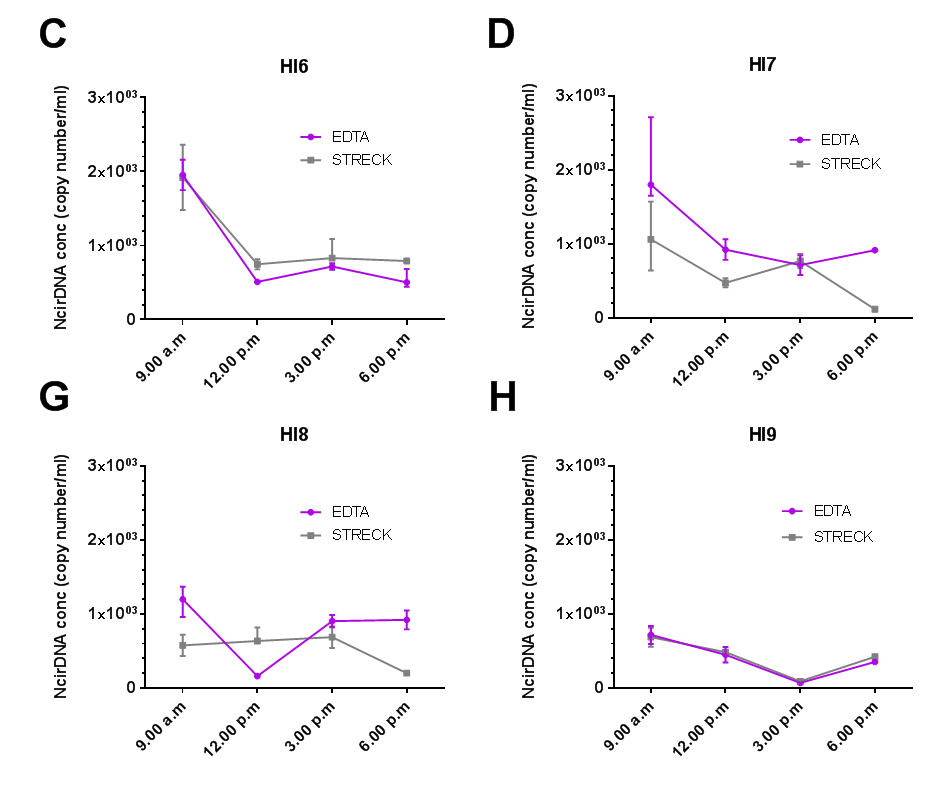


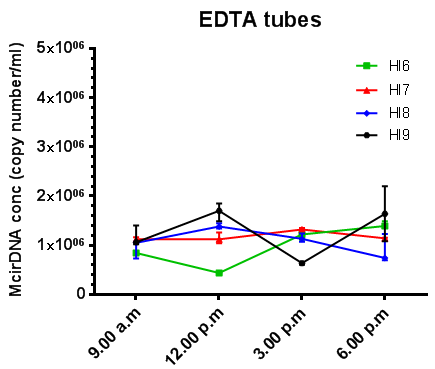

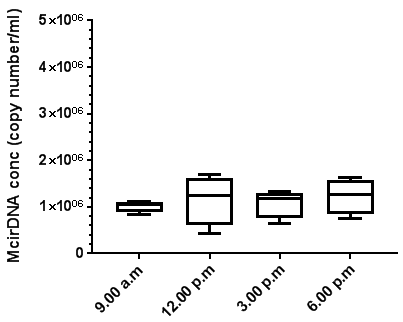


**A**

**B**

**Supplementary Figure S5: Effect of the blood collection time on McirDNA amount in healthy volunteers.** McirDNA concentration was determined exactly as described Methods section. Blood was collected in EDTA tubes. *(A)* Variation of McirDNA concentration expressed as copy number per ml for HI6, HI7, HI8 and HI9. Data represent triplicate median values with range. *(B)* Boxplot analysis. Boxplot represent median with min to max of values. Blue and yellow arrows indicate breakfast and lunch respectively.

**
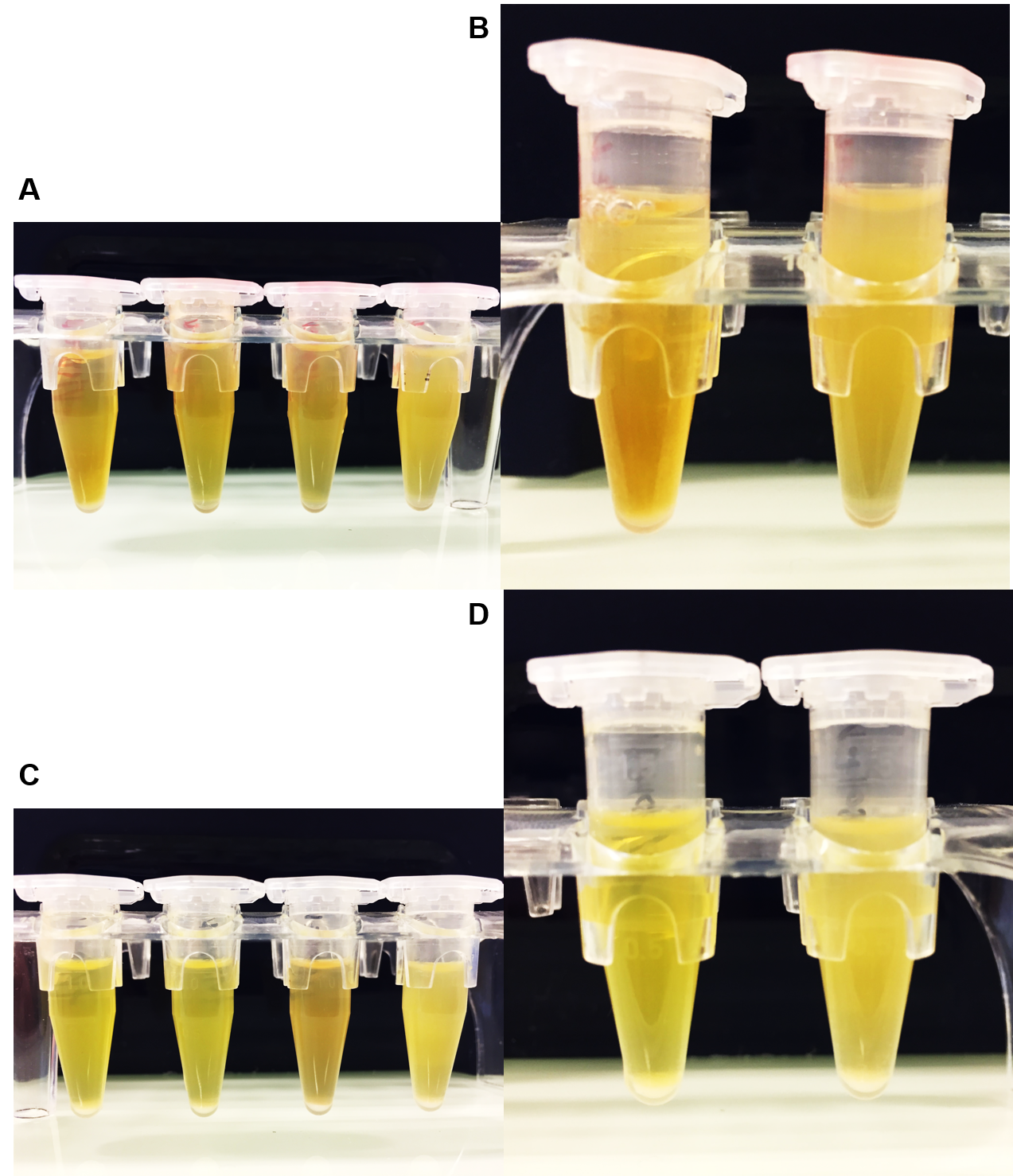
**

**Supplementary Figure S6: Effect of the collection time on the appearance of the plasma.** Images of the tubes containing plasma from the same healthy individual (HI6) collected at various time during a single day. *(A, B)* Plasma from blood collected in EDTA tubes. *(C, D)* Plasma from blood collected in BCT tubes. *(B, D)* Plasma from blood collected at 9:00 AM before food intake (on the left) and at 6:00 PM (on the right), respectively.

**Supplementary Figure S7: Potential influence of the menopause on NcirDNA concentration.** Boxplot analysis of NcirDNA concentration determined in the plasma of healthy (*A*) and mCRC females (*B*). The groups were dichotomized in 3 populations upon various age: < 45 year-old, 46-54 year-old and ≥ 55 year-old*.* Boxplot represent median with min to max of values and Kruskal-Wallis rank test was performed. A probability of ≤ 0.05 was considered to be statistically significant; * p ≤ 0.05, ** p ≤ 0.01, *** p ≤ 0.001, **** p ≤ 0.0001.

1

2

3

5

4

10

6

7

8

9

10 pg/µL

4 pg/µL

3 pg/µL

0.5 pg/µL

0.1 pg/µL

**Supplementary Figure S8: Quantification of increasing dilutions of WT *KRAS* human colorectal cell line DNA (DiFi) under Poisson law distribution.** In order to control accuracy of the cirDNA copy number, we performed experiments using Poisson law distribution on 10 replicate determinations of increasing DNA dilutions. Data revealed that the minimal dilution in which all replicates/wells are positive is 4 pg/µL (an average of about one copy per µl) and that the maximal dilution in which at most one replicate is positive corresponds to about one copy per 10 wells. Consequently, our Q-PCR systems enable the detection of a single copy of nuclear genome copy. In addition, this data suggests that the value of the total DNA concentration is accurate. Demonstration of the capacity in detecting a single DNA fragment molecule was previously described by targeting KRAS G12V mutant fragments^59^.

Spearman r = 0,882

P value < 0,0001

Spearman r = 0,762

P value < 0,0001

**Supplementary Figure S9: NcirDNA concentration determined by targeting both *KRAS* and *BRAF* sequences.** Scatterplot representation of NcirDNA concentrations as determined by targeting both *KRAS* and *BRAF* sequences, in healthy individuals (*A*) and mCRC patients (*B*), respectively. Spearman's rank correlation coefficient was performed. A probability of ≤ 0.05 was considered to be statistically significant; * p ≤ 0.05, ** p ≤ 0.01, *** p ≤ 0.001, **** p ≤ 0.0001.

**Supplementary Figure S10: Comparison of NcirDNA concentration between mCRC patients and healthy individuals.** Boxplot representation of NcirDNA concentrations as determined by targeting *BRAF* sequence. The cohort was dichotomized in two populations: mCRC patients (N=101) and healthy individuals (N=62). Boxplot represent median with min to max of values and Mann-Whitney U test was performed for comparison. A probability of ≤ 0.05 was considered to be statistically significant; * p ≤ 0.05, ** p ≤ 0.01, *** p ≤ 0.001, **** p ≤ 0.0001.

**Supplementary Table S1: Summary of NcirDNA and McirDNA quantification.** Mann-Whitney U tests were performed for analysis. A probability of ≤ 0.05 was considered to be statistically significant; * p ≤ 0.05, ** p ≤ 0.01, *** p ≤ 0.001, **** p ≤ 0.0001.

**Supplementary Table S2: Summary of the multivariate analysis.** Logistic regressions, with a stepwise selection procedure, were used to investigate known predictive factors. NcirDNA concentration in healthy individuals (*A*) and mCRC patients (*C*). McirDNA concentration in healthy individuals (*B*) and mCRC patients (*D*). A probability of ≤ 0.05 was considered to be statistically significant.

| **Pre-analytical considerations** | **Group** | **N** | **In accordance with previous works** |
| --- | --- | --- | --- |
| **NcirDNA concentration** | | | |
| Increased 2 days after blood draw in EDTA tubes (storage at +4°C) | Healthy volunteers | 5 | Barrett et al. (2011)  Zhang et al. (2008)  Chan et al. (2005) |
| Stable up to 7 days with BCT tubes  (storage at room temperature) | Healthy volunteers | 4 | Parpart-Li et al. (2017)  Warton et al. (2017)  Alidoutsy et al. (2017) |
| Influence of day-time of blood draw : Need of empty stomach at collection time | Healthy volunteers | 4 | **New observation** |
| **McirDNA concentration** | | | |
| Increased 2 days after blood draw in EDTA tubes (storage at +4°C) | Healthy volunteers | 5 | **New observation** |
| No influence of day-time of blood draw | Healthy volunteers | 4 | **New observation** |

**Supplementary Table S3: Summary of the observations made on the influence of pre-analytical factors.**

| **Sample** | **Mean Cq** | **Mean [DNA] (ng/µL)** | **Mean [DNA] (pg/µL)** | **Mean [DNA] (copies/µL)** | **Mean [DNA] (copies/well)** | **Expected [DNA] (copies/well)** |
| --- | --- | --- | --- | --- | --- | --- |
|  |  |  |  |  |  |  |
| DiFi 10 pg/µl | 33,23 | 0,007 | 7,07 | 2,14 | 10,71 | 14,00 |
| DiFi 4 pg/µl | 34,59 | 0,003 | 2,67 | 0,81 | 4,04 | 5,60 |
| DiFi 3 pg/µl | 35,53 | 0,0008 | 0,80 | 0,24 | 1,21 | 4,20 |
| DiFi 0,5 pg/µl | 35,96 | 0,0005 | 0,55 | 0,17 | 0,83 | 0,70 |
| DiFi 0,1 pg/µl | 37,66 | 0,0002 | 0,16 | 0,05 | 0,25 | 0,14 |

**Supplementary Table S4: Summary of the results obtained from the quantification of increasing dilutions of WT *KRAS* human colorectal cell line DNA (DiFi) under Poisson law distribution (Supplementary Figure S8).**
